# Complete genome sequences of colistin-resistant *Klebsiella pneumoniae* isolates from rectal swab samples of intensive care unit patients at a tertiary hospital in Kampala, Uganda

**DOI:** 10.1128/mra.00003-25

**Published:** 2025-03-25

**Authors:** Maximilian A.K. Magulye, Reuben S. Maghembe, Simon Sekyanzi, Beatrice Achan, Savannah Mwesigwa, Eric Katagirya

**Affiliations:** 1 Department of Immunology and Molecular Biology, School of Biomedical Sciences, College of Health Sciences, Makerere University 58588, Kampala, Uganda; 2 Department of Microbiology and Parasitology, Faculty of Medicine, Kairuki University, Dar es Salaam, Tanzania; 3 Department of Health and Biomedical sciences, Didia Education and Health Organization (DEHO), Shinyanga, Tanzania; 4 Omics and Bioinformatics Section, DABA Biotech. Ltd, Dar es Salaam, Tanzania; 5 Department of Microbiology and Parasitology, Faculty of Medicine, St. Francis University College of Health and Allied Sciences (SFUCHAS) 518753, Ifakara, Tanzania; 6 Department of Microbiology and Immunology, Faculty of Biomedical Sciences, Kampala International University - Western Campus 365672, Bushenyi, Uganda; 7 Department of Medical Microbiology, School of Biomedical Sciences, College of Health Sciences, Makerere University 58588, Kampala, Uganda; Department of Biology, Queens College, Queens, New York, USA

**Keywords:** *Klebsiella pneumoniae*, colistin resistance, genomes, whole genome sequencing

## Abstract

Hospital-acquired infections caused by multidrug-resistant *Klebsiella pneumoniae* are steadily increasing worldwide. Here, we report complete genome sequences of colistin-resistant *K. pneumoniae* isolates from rectal swab samples of intensive care unit (ICU) patients. Genome size in each strain varied with mean genome size of 5,335,766 bp and GC content averaging 57.48%.

## ANNOUNCEMENT

*Klebsiella pneumoniae* belongs to the family *Enterobacteriaceae* and is described as the Gram-negative, encapsulated, non-motile, rod shape and facultative anaerobic bacteria. In humans, *K. pneumoniae* normally forms part of the healthy human microbiome and is particularly concentrated in the gastrointestinal tract and a few in the nasopharynx (1). Despite being part of the normal microbiome, *K. pneumoniae* is responsible for a significant proportion of hospital-acquired infections. Worldwide reports reveal the increasing trend of multidrug-resistant *K. pneumoniae* in healthcare-associated infections with colistin-resistant *K. pneumoniae* among the documented notorious culprits, which is worrying (2). The complete genome sequences of multidrug-resistant *K. pneumoniae* strains will contribute to the understanding of its genetic characterization and give insights into colistin resistance in hospitalized patients. Fecal matter was collected by carefully inserting the swab into the anal canal about 1 in. (2.5 cm) beyond the anal sphincter, rotating it for about 10 s to sample the anal crypts before withdrawing it carefully. Rectal swabs were then suspended in 5 mL buffered peptone water and incubated overnight for 24 h at 37°C in ambient air. Aliquots of about 10 µL of the overnight growth were inoculated on MacConkey agar and incubated aerobically at 37°C for 2 4 h and colonies suggestive of *K. pneumoniae* were examined and cultured. From these cultures, identification of the *K. pneumoniae* isolates was carried out using conventional biochemical tests (3). Susceptibility testing was done using the Kirby–Bauer disk diffusion method and colistin broth microdilution according to the Clinical Laboratory Standard Institute (CLSI) guidelines (4). The DNA from a single colony of overnight culture of colistin-resistant *K. pneumoniae* (in Mueller Hinton Agar) was extracted using a Cetyltrimethylammonium bromide (CTAB) protocol as previously described by Willner and colleagues following manufacturer’s recommendations (5). For preparation of ready-to-sequence libraries of bacterial genomes, Nextera DNA XT library preparation kit (Illumina, San Diego, CA, USA) was used. The entire genome of the *K. pneumoniae* was sequenced on the Illumina Novaseq 6000 platform, which yielded 4 gb of 150 bp length-paired end reads per each sample. Raw sequenced reads were quality controlled using FastQC (v.11.5) and using Trimmomatic (v0.38); raw reads were trimmed, removing adaptors and low-quality sequences. The raw reads were *de novo* assembled into contigs using SPAdes v.3.15.5 (6), and assembly quality was assessed using BUSCO v5.4.7 (7). Chromosomes were generated by mapping the *de novo* assembly-derived contigs to relevant reference genomes using the MeDuSa pipeline (8) (https://github.com/arthurvinx/MEDUSA). Chromosomes were annotated using the NCBI Prokaryotic Genome Annotation Pipeline (PGAP) v6.6 (9). Default parameters were used for all software except where otherwise noted. The circular genome features of *K. pneumoniae* strains are summarized in Table 1.

**TABLE 1:** Genome features of *K. pneumoniae* strains

Strain	MAKM-RS045	MAKM-RS081	MAKM-RS083	MAKM-3381	MAKM-5490
Genome size (bp)	5,403,338	5,319,428	5,231,098	5,321,788	5,403,180
G + C (%)	57.46	57.35	57.61	57.58	57.40
No of Contigs	48	74	33	116	45
Contigs N50 (kb)	434.4	725.6	409	375.2	302.2
Completeness (%)	98.91	98.35	99.01	98.08	98.03
No. of genes	5,909	6,054	5,712	6304	5,901
CDSs (total)	5,818	5,961	5,622	6241	5,811
CDSs (with protein)	5,640	5,683	5,405	5790	5,582
No of tRNAs	79	75	74	74	74
GenBank accession numbers	CP130497.1	CP129536.1	CP129612.1	CP129540.1	CP130492.1
SRA accession numbers	SRR32280978	SRR32280977	SRR32280976	SRR32280975	SRR32280974

## Data Availability

*K. pneumoniae* whole genome raw sequence data were submitted to NCBI, GenBank under SRA number PRJNA985213.

## References

[1] Joseph L, Merciecca T, Forestier C, Balestrino D, Miquel S. 2021. From Klebsiella pneumoniae colonization to dissemination: an overview of studies implementing murine models. Microorganisms 9:1282. doi:10.3390/microorganisms9061282 34204632 PMC8231111

[2] Panigrahi K, Pathi BK, Poddar N, Sabat S, Pradhan S, Pattnaik D, Patro S, Praharaj AK. 2022. Colistin resistance among multi-drug resistant gram-negative bacterial isolates from different clinical samples of ICU patients: prevalence and clinical outcomes. Available from: 10.7759/cureus.28317 PMC9499824 36158344

[3] Mac Faddin JF. 1980. Biochemical tests for identification of medical. Williams & Wilkins Co. Available from: https://cir.nii.ac.jp/crid/1130000797102367104

[4] Wayne P. 2020. CLSI performance standards for antimicrobial susceptibility testing. In CLSI supplements M100

[5] Willner D, Daly J, Whiley D, Grimwood K, Wainwright CE, Hugenholtz P. 2012. Comparison of DNA extraction methods for microbial community profiling with an application to pediatric bronchoalveolar lavage samples. PLoS One 7:e34605. doi:10.1371/journal.pone.0034605 22514642 PMC3326054

[6] Bankevich A, Nurk S, Antipov D, Gurevich AA, Dvorkin M, Kulikov AS, Lesin VM, Nikolenko SI, Pham S, Prjibelski AD, Pyshkin AV, Sirotkin AV, Vyahhi N, Tesler G, Alekseyev MA, Pevzner PA. 2012. SPAdes: A new genome assembly algorithm and its applications to single-cell sequencing. J Comput Biol 19:455–477. doi:10.1089/cmb.2012.0021 22506599 PMC3342519

[7] Simão FA, Waterhouse RM, Ioannidis P, Kriventseva EV, Zdobnov EM. 2015. BUSCO: assessing genome assembly and annotation completeness with single-copy orthologs. Bioinformatics 31:3210–3212. doi:10.1093/bioinformatics/btv351 26059717

[8] Bosi E, Donati B, Galardini M, Brunetti S, Sagot M-F, Lió P, Crescenzi P, Fani R, Fondi M. 2015. MeDuSa: a multi-draft based scaffolder. Bioinformatics 31:2443–2451. doi:10.1093/bioinformatics/btv171 25810435

[9] Tatusova T, DiCuccio M, Badretdin A, Chetvernin V, Nawrocki EP, Zaslavsky L, Lomsadze A, Pruitt KD, Borodovsky M, Ostell J. 2016. NCBI prokaryotic genome annotation pipeline. Nucleic Acids Res 44:6614–6624. doi:10.1093/nar/gkw569 27342282 PMC5001611

